# Loss of Histone Locus Bodies in the Mature Hemocytes of Larval Lymph Gland Result in Hyperplasia of the Tissue in *mxc* Mutants of *Drosophila*

**DOI:** 10.3390/ijms21051586

**Published:** 2020-02-26

**Authors:** Masanori Kurihara, Kouyou Komatsu, Rie Awane, Yoshihiro H. Inoue

**Affiliations:** Department of Insect Biomedical Research, Center for Advanced Insect Research Promotion, Kyoto Institute of Technology, Matsugasaki, Sakyo-ku, Kyoto 606-0962, Japan; masa.megane.masa@gmail.com (M.K.); yoshikouyou@gmail.com (K.K.); r1a.awa@gmail.com (R.A.)

**Keywords:** tumorigenesis, hemocytes, Adgf-A, STAT, lymph gland, *Drosophila*

## Abstract

Mutations in the *multi sex combs* (*mxc*) gene in *Drosophila* results in malignant hyperplasia in larval hematopoietic tissues, called lymph glands (LG). *mxc* encodes a component of the histone locus body (HLB) that is essential for cell cycle-dependent transcription and processing of histone mRNAs. The mammalian *nuclear protein ataxia-telangiectasia* (*NPAT*) gene, encoded by the responsible gene for ataxia telangiectasia, is a functional Mxc orthologue. However, their roles in tumorigenesis are unclear. Genetic analyses of the *mxc* mutants and larvae having LG-specific depletion revealed that a reduced activity of the gene resulted in the hyperplasia, which is caused by hyper-proliferation of immature LG cells. The depletion of *mxc* in mature hemocytes of the LG resulted in the hyperplasia. Furthermore, the inhibition of HLB formation was required for LG hyperplasia. In the mutant larvae, the total mRNA levels of the five canonical histones decreased, and abnormal forms of polyadenylated histone mRNAs, detected rarely in normal larvae, were generated. The ectopic expression of the polyadenylated mRNAs was sufficient for the reproduction of the hyperplasia. The loss of HLB function, especially 3′-end processing of histone mRNAs, is critical for malignant LG hyperplasia in this leukemia model in *Drosophila*. We propose that *mxc* is involved in the activation to induce adenosine deaminase-related growth factor A (Adgf-A), which suppresses immature cell proliferation in LG.

## 1. Introduction

Leukemia encompasses a group of blood cancers that usually develop in the bone marrow and result in the production of excessive abnormal blood cells [1,2,3]. Mutations in many genes that are responsible for the pathogenesis of leukemia have been identified via genome analysis of patient-derived leukemic cells [4,5,6]. Recent studies have attempted to identify and characterize new genes that are involved in leukemia and related diseases. Previous studies have indicated that mutations in *NPAT* are related to a class of malignant lymphomas, called Hodgkin’s lymphoma [7,8]. This gene is also involved in ataxia disorder, which is associated with cancer-related symptoms [9]. *NPAT* encodes a nuclear protein that plays an essential role in cell cycle progression to the S phase [10,11]. NPAT/p220 is a transcription factor that controls cell cycle-dependent histone gene transcription in an E2F-dependent manner [12]. The protein is phosphorylated by cyclin E-Cdk2, which stimulates histone mRNA synthesis [13,14,15]. However, the mechanisms underlying the alteration of expression and/or function of the proteins that eventually led to tumorigenesis of hematopoietic cells are still not known.

*Multi sex combs (mxc),* a *Drosophila* gene encoding a 1837 amino acid-long protein possessing a region that encompasses the lissencephaly homology (LisH) domain, shows high amino acid sequence similarity to human NPAT [16,17]. This gene was previously identified as a member of the Polycomb group genes and it has been shown to function as a tumor suppressor in *Drosophila* on the basis of mutant phenotypes [18,19]. In hemizygous mutant larvae for *mxc^mbn1^*, which is a lethal allele of the gene, a hyperplasia phenotype appeared in the lymph gland (LG) (a specialized hematopoietic organ) at the larval stage. Previous reports demonstrated that the mutant larvae possess enlarged lymph glands and the mutant cells invaded the surrounding tissues when these cells were injected into normal adult abdominal cavities [20,21,22]. Extra hemocytes, as well as abnormally differentiated hemocytes, were observed in the hemolymph of the mutant larvae [18,20,22,23]. Conversely, the larval imaginal discs were underdeveloped in the *mxc* mutants. The development of germ line cells, including the progression of meiotic divisions, was also compromised in the *mxc* mutants [19,22,24,25].

*Drosophila* hemocytes are responsible for immune response, such as phagocytosis and protection from infectious bacteria and foreign matter [26]. The circulating hemocytes arise from two distinct hematopoietic tissues, namely the embryonic head mesoderm and the LG at the later larval stage [27,28,29]. The LG contains hematopoietic progenitor cells called pro-hemocytes, which can give rise to three types of hemocytes: plasmatocytes, lamellocytes, and crystal cells [27,30,31]. During the larval stage, precursor cells that differentiate into hemocytes are generated, which undergo several rounds of proliferation in the LGs. The formation of the hematopoietic tissue is completed in the mature stage of the third instar larvae. The LG that develops under normal conditions has a paired multi-lobed structure in the third instar larvae. The lobes are clusters of hematopoietic cells arranged in a hemispheric pattern segmentally aligned in pairs along the anterior-posterior (A-P) axis of the tissue. They consist of a pair of larger primary (first) lobe at the anterior end, several successive series of secondary (second) lobe, and a tertiary lobe can be observed in this order [23,32,33]. The first lobe consists of three regions: the cortical zone (CZ), which has abundant mature hemocytes; the medullary zone (MZ), which is formed by the immature precursor cells of hemocytes located inside the lobe; the Posterior Signaling Center (PSC), which is a small group of cells that reside on the most posterior region of the first lobe adjacent to the second lobe [30]. The PSC cells play critical roles as hematopoietic stem cell niche. They also play a central role in regulation of proliferation and differentiation of immature cells in the LG [34,35]. The role of the PSC in controlling homeostasis of hemocytes in *Drosophila* is reminiscent of the hematopoietic stem cell niche in the bone marrow of mammals. Pvf1, an extra-cellular protein that is secreted from PSC, binds to its receptor, Pvr, which is localized on the surface of mature hemocytes in the CZ. Subsequently, the binding activates the STAT-mediated signaling pathway that is downstream of the receptor and it eventually induces the expression of Adgf-A (adenosine deaminase-related growth factor-A). This factor, which is synthesized in CZ, suppresses the proliferation of immature cells in the MZ of the first lobe and those consisting of the posterior lobes [36,37].

Mxc is a component of the histone locus body (HLB), which is localized on a single nuclear foci that corresponds to a histone gene cluster in the chromatin [13,16,38]. The HLB is essential for DNA replication-dependent expression of five canonical histones, namely, histoneH1, H2A, H2B, H3, and H4, as well as for the processing of the histone mRNAs [39,40,41]. Among the HLB components, the Spt6 and Mute proteins are required for the transcription of the histone genes. In contrast, the ribonucleoprotein (RNP) containing the U7 snRNA, FLASH, and Cpsf family proteins play key roles in mRNA processing [39,42,43,44]. The proto-HLB complex containing Mxc and FLASH are initially assembled and recruited to a histone gene cluster on the genome. Subsequently, additional components, U7snRNP and Mute, are assembled on the proto-HLB complexes. At the onset of S phase, Spt6 and Simplekin are further added to generate a mature HLB complex on the histone gene cluster [45]. The mature complex is involved in both stimulating the transcription of the five canonical histone genes and RNA processing at the 3′ end, so as to generate unique mRNA structures lacking poly(A) tails [16,17,41].

Few studies have investigated why the *mxc* mutant larvae resulted in malignant hyperplasia in the *Drosophila* hematopoietic tissue in the larval stage. In this study, we aimed to investigate the mechanism via which malignant LG hyperplasia occurs in the *mxc* mutants. Overall, we propose that the loss of HLB in the *mxc^mbn1^* flies resulted in the LG malignant hyperplasia phenotype. Our findings in the *Drosophila* leukemia model will enable us to consider a similar involvement of NPAT, the human counterpart of Mxc, in the pathogenesis and development of leukemia. Our observations indicate that the disruption of the regulatory mechanism that is required for maintaining undifferentiated blood cell precursors in a quiescent state might be involved in leukemia pathogenesis.

## 2. Results

### 2.1. Loss of Function Mutations of mxc and Its Depletion in LG Resulted in Hyperplasia Showing Increased Ratio of Immature Hemocytes in the Larval Tissues

**Figure 1:**
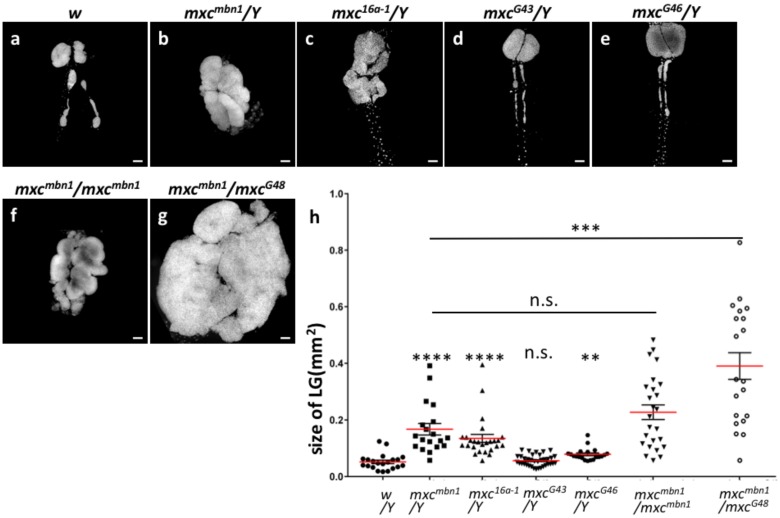
*mxc^mbn1^* and *mxc^16a-1^* showing a hyperplasia phenotype of larval lymph glands (LGs) are hypomorphic and recessive alleles of *mxc* gene. (**a**–**g**) Fluorescence micrographs of DAPI-stained LGs prepared from third instar larvae at mature stage. (**a**) A LG from control male larva (*w/Y*), (**b**) a larva hemizygous for *mxc^mbn1^* (*mxc^mbn1^*/*Y*), (**c**) a larva hemizygous for *mxc^16a-1^* (*mxc^16a-1^*/*Y*), (**d**) a larva hemizygous for a viable but male and female sterile allele, *mxc^G43^* (*mxc^G43^*/*Y*), (**e**) a larva hemizygous for a less sever pharate adult lethal allele, *mxc^G46^* (*mxc^G46^*/*Y*). (**f**,**g**), A LG from a female larva homozygous for *mxc^mbn1^* (*mxc^mbn1^/mxc^mbn1^*) (**f**), a trans-heterozygote between *mxc^mbn1^* and amorphic allele, *mxc^G48^* (*mxc^mbn1^*/*mxc^G48^*) (**g**). (**h**), Quantification of LG size from mature third instar larvae with each genotype. Significant differences from control (*w*) are indicated by **** *p* < 0.0001, *** *p* < 0.001, ** *p* < 0.01. Student’s *t*-test was used for comparisons between control (*w*) and *mxc^G46^/Y*. Welch’s *t*-test was used for other five comparisons. Bar: 100 μm. Red bars indicate average size of LG and error bars are SEM.

The *mxc^mbn1^* mutant larvae showed malignant hyperplasia of hematopoietic tissue, called LGs (see Introduction). Another recent study revealed that an over-proliferation of immature cells, but not mature cells, occurred in the most anterior lobes of LGs of *mxc^mbn1^* larvae [23]. In this study, we further investigated whether the development of LG hyperplasia in the LGs was affected in the hemizygotes for lethal alleles. We confirmed that the remarkable over-growth of the lobes located at the most anterior side of the LG occurred in the mature larvae of the hemizygotes for two *mxc* alleles, *mxc^mbn1^* and *mxc^16a-1^* (Figure 1a–c). The average size of LGs from the mature stage of the third instar larvae of *mxc^mbn1^* was >3.3-fold larger than that of the control (*w*) at the same stage (Figure 1b,h, *n* = 19, *p* < 0.0001 in Welch’s *t*-test). *mxc^16a-1^* also possessed 2.7-fold larger LGs than that of the control (Figure 1c,h, *n* = 26, *p* < 0.0001). The LG hyperplasia in *mxc^16a-1^* was less severe than that in the *mxc^mbn1^* larvae. Hemizygotes for a viable but male-female sterile allele, *mxc^G43^,* did not show the LG hyperplasia phenotype (Figure 1d,h), while hemizygotes for a less severe hypomorphic allele, *mxc^G46^*, died at the pharate adult stage and showed weak LG hyperplasia phenotype (1.5-fold larger LG on average, *p* < 0.01 in Student’s *t*-test) (Figure 1e,h). The largest lobe, called the first lobe, is located at the most anterior side of LG. After the first lobe, the secondary lobe and the tertiary lobe are aligned in a row toward the posterior, with insertion of single pericardial cells (PC) in between (Supplementary Figure S1a). The hyperplasia in *mxc^mbn1^* larvae was initially detected in most anterior lobes originally located in the first lobes of the third instar larvae in the earlier stage (Supplementary Figure S1b), and subsequently, it became prominent in the more posterior lobes (Supplementary Figure S1c,d). In the mature wandering larvae, the lobes were hard to distinguish, as the severity of LG disintegration increased with development (Figure 1b–d). We have frequently observed the LGs in these mutants, in which the first lobes appear to be lost (11 out of 11 larvae in the early third instar stage), while the largest lobes corresponding to the first lobes were located at the anterior end of the LGs. This phenotype was similar to that previously reported in other LG hyperplasia mutants (Karamarz et al., 2012). Although then PC cells are originally localized between lobes in normal LGs (Supplementary Figure S1a’), they are positioned at the anterior end of the most enlarged lobe in both hemispheres of the mutant LG. A part of the original first lobe consisting of some DAPI-stained cells remained at the anterior side of the PCs (Supplementary Figure S1b’). These LG phenotypes showing abnormal lobe organization suggested that the first lobes that were originally located at the anterior end of the LG had disintegrated after overgrowth of the lobes in the *mxc* mutants. We realized in those mutants that the LG hyperplasia could occur in more posterior lobes, as well as in the anterior end of the LGs (Figure 1b,c and Supplementary Figure S1b–d).

In normal LGs, the first lobe consists of three regions, namely the CZ enriched with mature hemocytes (Supplementary Figure S2a”), the PSCs, which functions as a stem cell niche (Supplementary Figure S2c”), and the MZ rich in immature precursors (Supplementary Figure S2b”). The posterior lobes of normal LG were made of only immature precursor cells. Interestingly, we observed that the most anterior lobe of the LG lacking the first lobe possessed mature hemocytes (Supplementary Figure S2d) and cells expressing a PSC cell marker (Supplementary Figure S2f) with immature precursors (Supplementary Figure S2e). These observations suggested that *de novo* production of PSC and CZ could occur in the case of a loss or dysfunction of the most anterior lobes.

### 2.2. Hemizygotes for Recessive Lethal But Not Amorphic Mutations of mxc Exhibited Hyperplasia of Lobes in Their Larval LGs

The LG phenotypes of *mxc^mbn1^* and *mxc^16a-1^* mutants were completely rescued by expressing GFP-tagged Mxc, which can rescue the lethal phenotype of the amorphic allele, *mxc^G48^* [16]. This suggested that both *mxc* mutations on the X-chromosome were recessive mutations, but not dominant oncogenic mutations. The severity of the LG hyperplasia phenotype increased with reduction in the gene dose of the *mxc* mutations. The average LG size of the mature third instar larvae of *mxc^mbn1^* was significantly larger than that of control (*w*) (Figure 1b,h). On the other hand, hemizygotes for the amorphic allele *mxc^G48^* died in the first larval stage. The LG hyperplasic phenotype was not observed in the *mxc^G48^* larvae at this stage. Furthermore, the trans-heterozygous females for *mxc^mbn1^* over the amorphic *mxc^G48^* allele showed more severe LG phenotypes (7.8-fold increase in LG size) than *mxc^mbn1^* homozygotes (4.4-fold increase in LG size) (*p* < 0.001, in Welch’s *t*-test) (Figure 1f–h). The results that are presented in Figure 1a,b,f–h indicated that these *mxc* mutations were hypomorphic but not amorphic. Overall, our observations indicated that the malignant hyperplasia phenotype results from a reduction, but not complete loss of function, of *mxc*.

### 2.3. Depletion of mxc in the Matured Hemocytes of LG Resulted in the Hyperplasia Phenotype of LG

**Figure 2:**
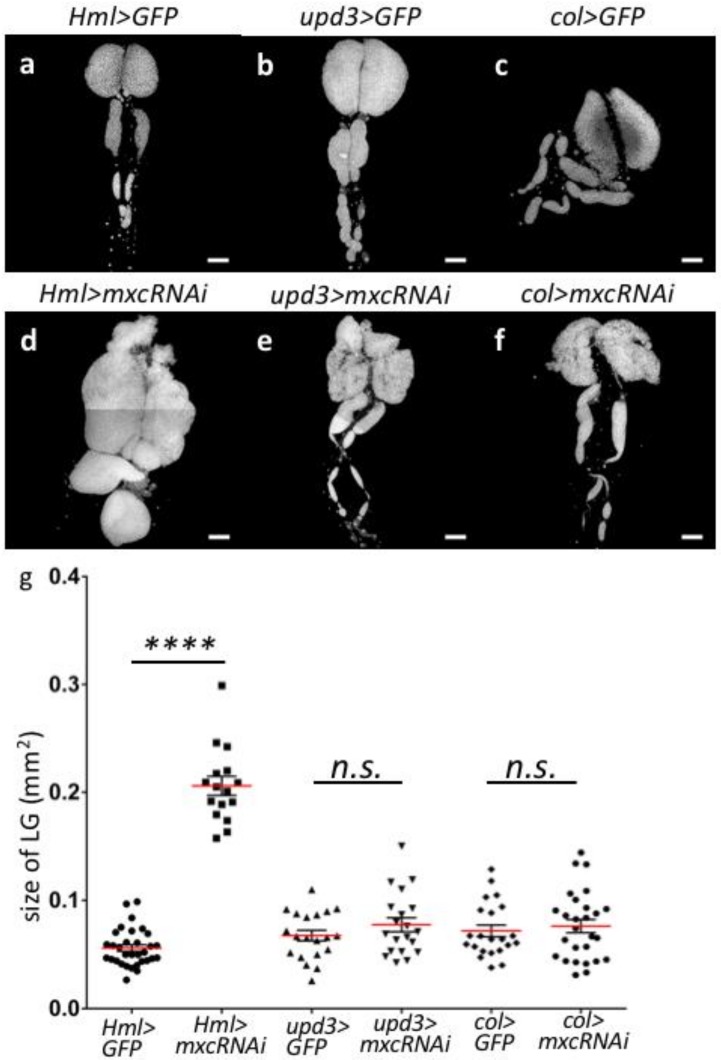
LG region-specific depletion of *mxc* reproduced the LG hyperplasia as observed in *mxc^mbn1^* larvae. (**a**–**f**) DAPI-stained LGs prepared from matured third instar larvae. (**a**–**c**) LGs having region-specific expression of GFP in CZ (*Hml>GFP*) (**a**), medullary zone (MZ) (*upd3>GFP*) (**b**), Posterior Signaling Center (PSC) (*col>GFP*) (**c**). (**d**–**f**) LGs having region-specific depletion of *mxc* in CZ (*Hml>mxcRNAi*) (**d**), MZ (*upd3>mxcRNAi*) (**e**), PSC (*col>mxcRNAi*) (**f**). (**g**) The sizes of LGs having *mxc* depletion in each of three different regions consisting of a LG. Bar: 100 μm. **** *p* < 0.0001 by Welch’s *t*-test. n. s.; not significant.

A previous study demonstrated that the immature precursor cells in the MZ of LG hyper-proliferate in the malignant hyperplasic LGs of *mxc^mbn1^* larvae [23]. In this study, we showed that *mxc^mbn1^* and *mxc^16a-1^* flies showing LG hyperplasia harbored recessive and severe hypomorphic *mxc* mutations. We investigated whether *mxc* depletion in LG reproduces the same hyperplasic phenotype to further confirm that the loss of *mxc* was responsible for LG hyperplasia. We induced the ectopic expression of dsRNA against *mxc* mRNA in the three regions of LG, namely, CZ, MZ, and PSC, while using region-specific Gal4 drivers. The *UAS*-*mxcRNAi^HMS00444^* used in this study can efficiently deplete endogenous *mxc* mRNA in the testes while using the testis-specific Gal4 driver [25]. In this study, we used the *Hml-Gal4* driver to deplete the endogenous mRNA in CZ, the *upd3-Gal4* driver to deplete it in MZ, and the *col-Gal4* driver for depletion in PSC (for control see Figure 2a–c; for *mxc* depletion see Figure 2d–f). We have not recognized significant differences among the GFP intensity in each nucleus of the control LG cells (*Hml>GFP*, *Upd>GFP* and *col>GFP*). Consequently, the average size of LG from mature larvae harboring CZ-specific depletion of the *mxc* mRNA (*Hml>mxcRNAi*) was significantly larger (3.5-fold) than that of the controls (*Hml>GFP*) (*n* = 16, *p* < 0.0001, in Welch’s *t*-test). In contrast, the average size of LGs in the larvae harboring MZ-specific depletion or that in the larvae with PSC-specific depletion did not significantly change (*n* > 20 in either comparison, *p* > 0.05, not significant in Welch’s *t*-test). In summary, the depletion of *mxc* mRNA in CZ resulted in a remarkable reproduction of the LG hyperplasia phenotype in the *mxc^mbn1^* larvae, while depletion in MZ or PSC did not significantly alter LG size (Figure 2g). Thus, these genetic data are consistent with the conclusion that the malignant *mxc* mutation is a loss of function mutation, but not a dominant neomorphic mutation. Furthermore, we concluded that reduction in the expression in CZ, but not in MZ or PSC, is mainly required for LG hyperplasia in *mxc^mbn1^* larvae.

A previous quantitative reverse transcription-polymerase chain reaction (qRT-PCR) experiment indicated that the mRNA level of *mxc* was not significantly altered in the *mxc^mbn1^* larvae [23]. Consistently, the results of published comprehensive RNA sequence analysis also showed that the ratio of the mRNA levels of *mxc* in *mxc^mbn1^* to that in a wild-type strain was 1.55 [23]. We next determined the DNA sequence of over 6.7 kb genomic region of the mutant gene, from 263 bp of the 5′ untranslated region (UTR) upstream of the ATG codon to 460 bp of the 3′UTR downstream of the stop codon, which included eight exons and seven introns, to understand the molecular features of the malignant *mxc^mbn1^* mutation. We detected two point mutations, A662V and T1321A, which cause amino acid substitutions, while no deletions or insertions were detected in the introns, 5′UTR, 3′ UTR, or introns (Supplementary Figure S3). T1321A is a nonsynonymous amino acid substitution, while A662V is a synonymous substitution. However, it is uncertain whether these mutations are responsible for the hyperplasia of LG in the *mxc^mbn1^* mutant larvae. We confirmed a previously reported result that the *mxc^16a-1^* mutant harbored a nonsense mutation, which produces a truncated polypeptide lacking residues after the 1824^th^ amino acid and containing an additional 31 amino acids at the C-terminal end [16]. Whether the truncated protein is responsible for the malignant phenotype has not been clarified.

### 2.4. HLB Formation Was Disrupted in Nuclei of LG Cells From mxc^mbn1^ Larvae

**Figure 3:**
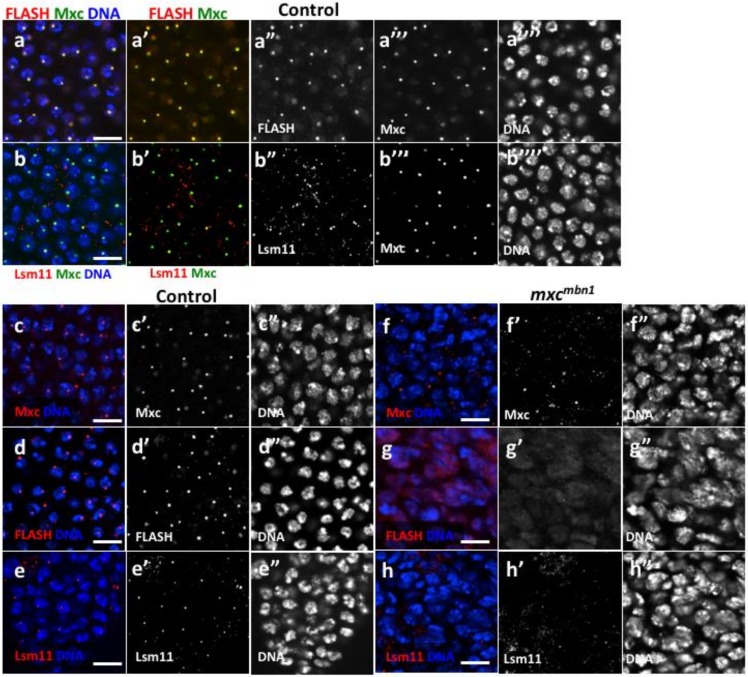
Histone locus body (HLB) formation was disrupted in LGs of *mxc^mbn1^* larvae. (**a**,**b**) Immunostaining of LG cells prepared from control third instar larvae expressing GFP-tagged Mxc (green in **a**, **b** and **a’**, **b’**, while in **a’’’** and **b’’’**) with antibodies against another HLB component, FLASH (**a**) or Lsm11 (**b**) (red in **a**, **b** and **a’**, **b’**, white in **a”** and **b”**). DAPI-staining (blue in **a** and **b**, white in **a’’’** and **b’’’**). Mxc-GFP foci are overlapped with FLASH foci and Lsm11 foci, indicating that these three components construct a single nuclear body, HLB. (**c**–**h**) Immunostaining of LG cells prepared from control third instar larvae with antibody against Mxc, FLASH or Lsm11. LG cells from wild-type (**c**–**e**) or from *mxc^mbn1^* mutant larvae (**f**–**h**). Anti-Mxc immunostaining (red in **c** and **f**, white in **c’** and **f’**), anti-FLASH immunostaining (red in **d** and **g**, white in **d’** and **g’**), and anti-Lsm11 immunostaining (red in **e** and **h**, white in **e’** and **h’**). Mxc foci, FLASH foci, or Lsm11 foci fails to be observed on chromatins in LGs from *mxc^mbn1^* larvae. DAPI-staining (**e”**–**h”**). Bar, 10 µm.

We next investigated whether the *mxc^mbn1^* mutation affected HLB formation in LG cells, as the gene product of *mxc* is a part of the nuclear body called HLB. The expression of Mxc-GFP in larval LG cells enabled the visualization of HLB containing Mxc at a single site on chromatin (Figure 3a,b). The HLB foci likely corresponded to the histone gene cluster, as shown in early embryonic cell nuclei [16,38]. These single Mxc foci were also visualized via immunostaining with anti-Mxc antibodies [16]. FLASH co-localizes to the histone locus on chromatin with Mxc, and together these proteins initiate the hierarchical assembly of HLB. Anti-FLASH immunostaining of normal cells showed that FLASH co-localized with Mxc in the HLB (Figure 3a,3a’). Anti-Lsm11 immunostaining further demonstrated that Lsm11, which is another known component that is subsequently recruited on the Pro-HLB, co-localized with Mxc in the HLB (Figure 3b). In contrast, Mxc foci that were detected by anti-Mxc immunostaining were greatly reduced in the LG nuclei from *mxc^mbn1^* larvae. (Figure 3c,f). Neither FLASH foci nor Lsm11 foci were observed in the LG nuclei of *mxc^mbn1^* mutant larvae (Figure 3g,h). From these immunostaining data, we concluded that HLB formation was disrupted in the LG cell nuclei of *mxc^mbn1^* larvae.

### 2.5. Inhibition of HLB Formation in CZ of Larval LGs Reproduced Hyperplasia of the Tissues

**Figure 4:**
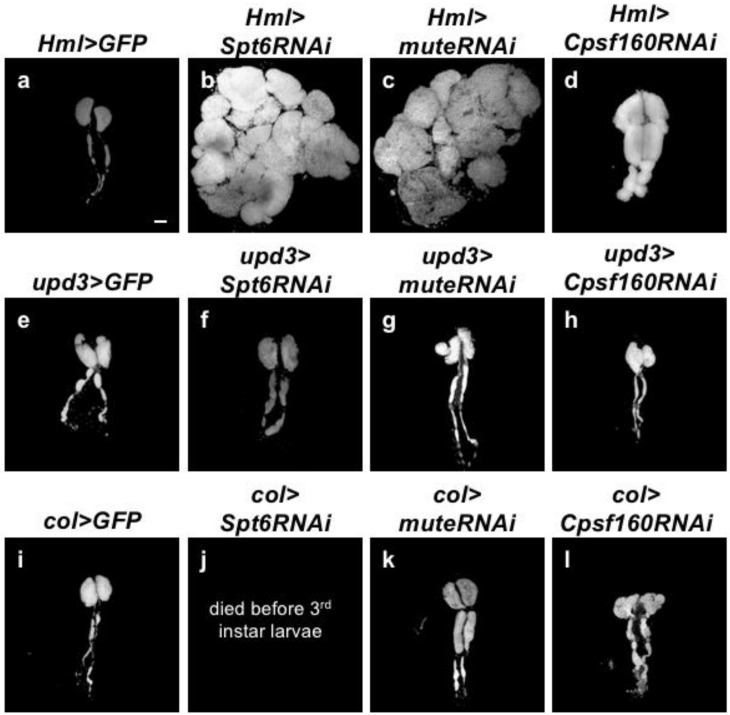

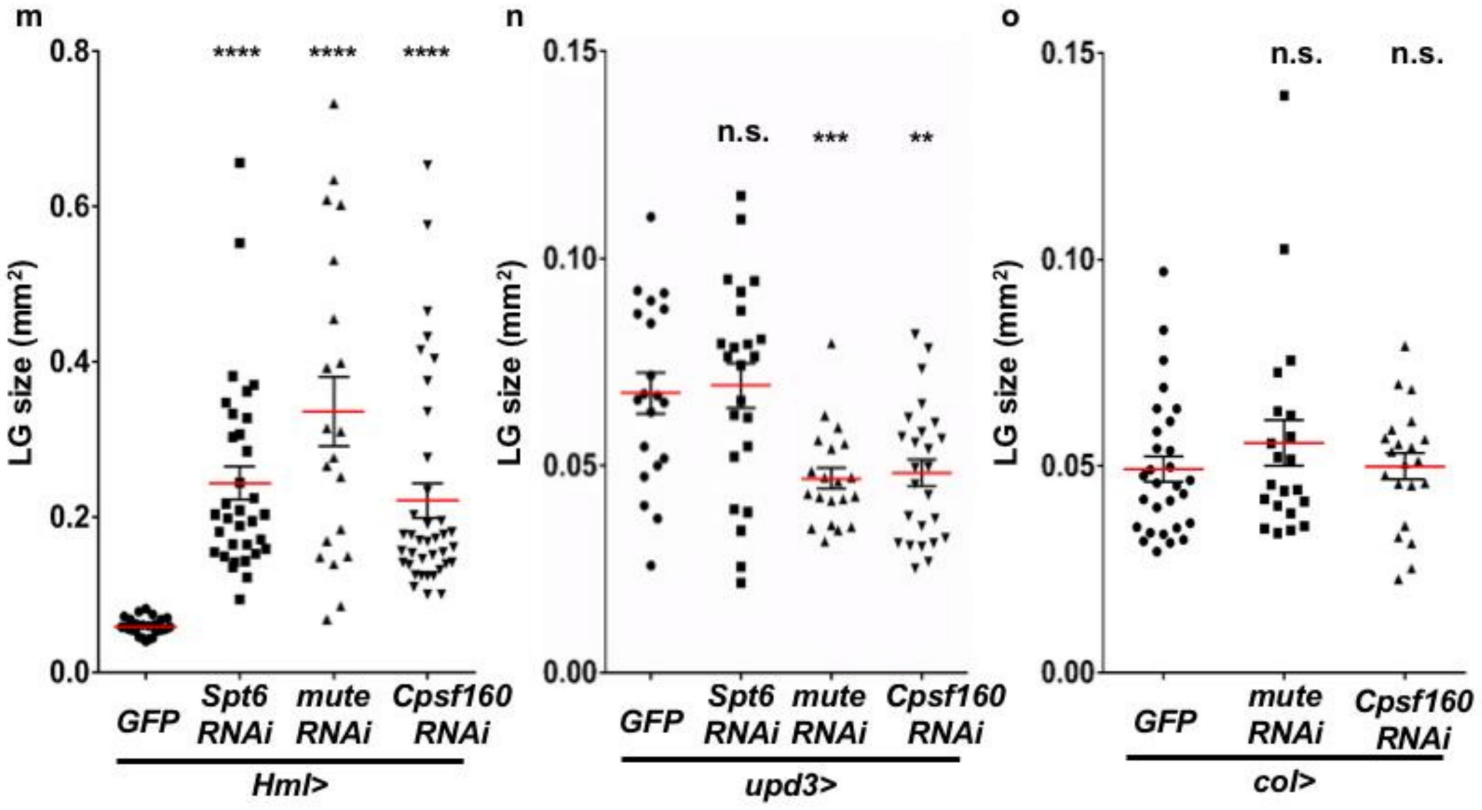
Region specific-depletion of HLB components in larval LG and their effects on reproduction of LG hyperplasia. (**a**–**i**,**k**,**l**) DAPI-stained LGs prepared from mature larvae at third instar stage. (**a**–**d**) LGs having ectopic expression in mature hemocytes contained abundantly in CZ of LG using *Hml-Gal4*. (**e**–**h**) LGs having ectopic expression in immature precursor cells enriched in MZ using *upd3-Gal4*. (**i**,**k**,**l**) LGs having ectopic expression in PSC cells using *col-Gal4*. (**j**) As *col>Spt6RNAi died* before third instar larvae, LGs at the same stage were not able to observe. (**a**,**e**,**i**) Control LGs expressing GFP in CZ (*Hml>GFP*) (**a**), MZ (*upd3>GFP*) (**e**) and PSC (*col>GFP*) (i). (**b**,**f**) LGs depleted of *Spt6* in CZ (*Hml>Spt6RNAi*) (**b**), MZ (*upd3>Spt6RNAi*) (**f**). (**c**,**g**,**k**) LGs depleted of *mute* in CZ (*Hml>muteRNAi*) (**c**), MZ (*upd3>muteRNAi*) (**g**) and PSC (*col>muteRNAi* (**k**). (**d**,**h**,**l**) LGs depleted of *Cpsf160* in CZ (*Hml>Cpsf160RNAi*) (**d**), MZ (*upd3>Cpsf160RNAi*) (**h**) and PSC (*col>Cpsf160RNAi*) (**l**). Bar: 100 μm. (**m**,**n**,**o**) Quantification of LGs that were prepared from mature third instar larvae with each genotype. ** *p* < 0.01, *** *p* < 0.001, and **** *p* < 0.0001 in Student *t*-test. Note that a significant LG hyperplasia, as observed in the *mxc* mutants, was commonly seen in mature hemocyte-specific depletion of all of four HLB components, including Mxc.

Genetic evidence showing that the formation of HLB was disrupted in *mxc^mbn1^* larvae and that *mxc* depletion in the CZ resulted in hyperplasia of the tissue suggested that the loss of HLB in the LG region might be responsible for LG hyperplasia. Hence, we performed depletion experiments to investigate whether a reduction of other components of HLB can reproduce LG hyperplasia. Among the HLB components, we selected two components that were involved in the transcription of canonical histone genes, namely, Spt6 and Mute. Additionally, we also selected Cpsf160, which is involved in processing of histone mRNAs. First, we confirmed that the induced expression of dsRNA against the mRNA of each component resulted in the efficient depletion of the corresponding mRNAs while using qRT-PCR analysis. The results of qRT-PCR using total RNAs from larvae ubiquitously expressing dsRNAs against the relevant mRNAs showed that the levels of the endogenous *mute* and *Cpsf160* mRNAs efficiently decreased to 68% and 43% of the control mRNA levels, respectively (*Act>muteRNAi* and *Act>Cpsf160RNAi*). As all these larvae raised at 28 °C died before the third instar stage, we isolated RNA from the larvae raised at 25 °C. The *mute* and *cpsf160* mRNAs in the LG of larvae raised at 28 °C (*Hml>muteRNAi*, *Hml>cpsf160RNAi*) were also efficiently depleted. As all the larvae with ubiquitous expression of dsRNA against *Spt6* mRNA, even those raised at 25 °C, died before the third instar stage, we were unable to isolate enough total RNA to confirm whether the *UAS-Spt6RNAi* stock can deplete the endogenous mRNA efficiently. It is highly possible that the lethality of *Act>Spt6RNAi* is due to a depletion of the *Spt6* mRNA in the larvae, as no off-targets against the dsRNA sequences expressed by the UAS-RNAi construct have been identified. Subsequently, we observed the LGs from mature third instar larvae, in which *Spt6* was depleted in CZ, MZ, and PSC while using three different *gal4* drivers; *Hml-Gal4, upd3-Gal4,* and *col-Gal4*, respectively (Figure 4a–i,k,l). The average LG size of the mature larvae with the depletion of *Spt6* mRNA in CZ (*Hml>Spt6RNAi*) increased by 3.5-fold when compared to that of control larvae (*Hml>GFP*) (*p <* 0.0001, Student’s *t*-test) (Figure 4m). We also observed abnormal lobe organization, absence of the original first lobes, enlargement of lobes located at the anterior end, as well as more posterior lobes. These LG phenotypes were similar to the phenotypes in *mxc^mbn1^*. In contrast, when compared to that in the control (*upd3>GFP*), the depletion of *Spt6* in MZ (*upd3>Spt6RNAi*) did not cause any significant alteration, (*p* > 0.05) (Figure 4e,f). Unexpectedly, the larvae with *Spt6* depletion in PSC died before the third instar stage. This might be responsible for the depletion effect in other tissues rather than in LG by *col-Gal4*. Consistently, the depletion of *mute* in CZ resulted in remarkable hyperplasia (more than five-fold larger than the size of the control) of the tissue, which indicated that the depletion of *mute* could reproduce the LG hyperplasia phenotype (Figure 4c,m). Conversely, when compared to the control, the depletion of *mute* and *cpsf160* in MZ suppressed LG growth by 40% (*p <* 0.001) (Figure 4g,h,n). However, neither *mute* nor *cpsf160* depletion in PSC showed any detectable difference in LG size and morphology, including lobe organization (Figure 4k,l,o). In summary, the depletion of the mRNAs of the four components of HLB, including Mxc in CZ, commonly resulted in the reproduction of LG hyperplasia, but not in MZ or PSC. Together with these genetic results, we concluded that the reduction of HLB in the CZ of the LG is responsible for the LG hyperplasia that was observed in *mxc^mbn1^* larvae.

### 2.6. Reduced mRNA Levels of Canonical Histone mRNAs and Production of Abnormal Polyadenylated mRNAs in mxc^mbn1^ Larvae

**Figure 5:**
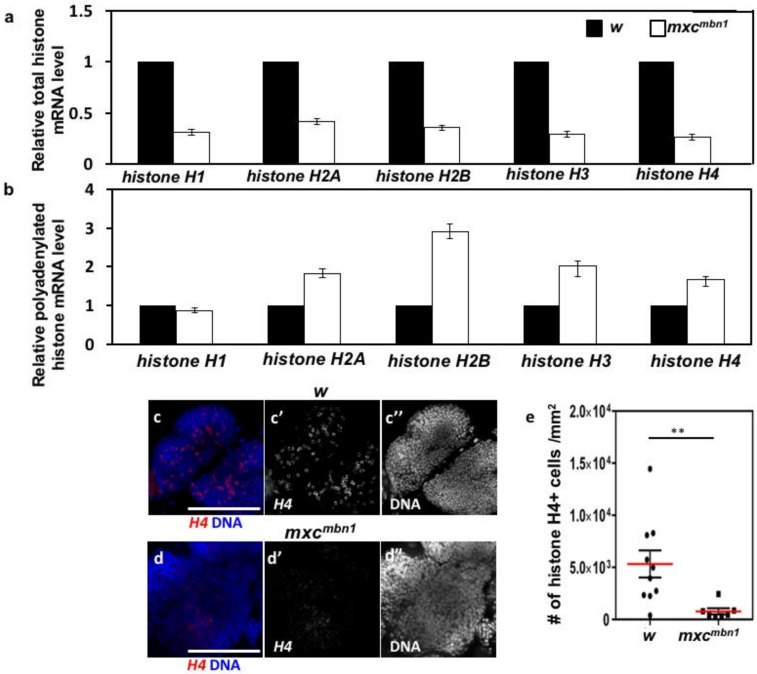
Quantification of mRNAs for five canonical histone genes in third instar larvae by qRT-PCR and in situ hybridization of *histone H4* mRNA in LG cells prepared from control and *mxc^mbn1^* larvae. (**a**) Relative levels of total mRNAs for five canonical histones, histoneH1, H2A, H2B, H3, H4 in control and *mxc^mbn1^* third instar larvae. Relative mRNA levels of the genes were calculated and normalized to the control level, which was set to 1.0 (*w/Y*) (** *p* > 0.01, Student’s *t*-test). qRT-PCR analysis to quantitate the amount of mRNA of each gene was repeated three times. Error bars represent s.d. Note that mRNA level of every canonical histone gene was reduced in amount, as compared with that of control. (**b**) Relative levels of polyadenylated mRNAs for five canonical histones, histone H1, H2A, H2B, H3, and H4 in control (*w*) as well as *mxc^mbn1^* third instar larvae. Note that mRNA level of every canonical histone gene except *histone H1* increased in amount. (**c**,**d**) RNA in situ hybridization of *histone H4* in LG cells prepared from male larvae at third instar larval stage. (**c**) control (*w/Y*), (**d**) *mxc^mbn1^*. (**e**) A comparison of LG cells showing signals for *histoneH4* mRNA (red in **c** and **d**, white in **c’** and **d’**). Note that a weaker in situ signal appeared in LGs of *mxc^mbn1^* and that less than one fifth of cells expressing *histone H4* mRNA can be observed in the mutant LGs. (**c”**,**d”**) DAPI-staining. Bar: 100 µm. ** *p* < 0.01 Student *t*-test.

The HLB is essential for both transcription of canonical histone genes and mRNA processing to produce mature mRNAs without poly(A) tails. Therefore, we next investigated whether the expression of the five canonical histone genes encoding histones H1, H2A, H2B, H3, and H4 were altered while using qRT-PCR (Figure 5a,b). The total RNAs were isolated from third instar larvae of control (*w*) and *mxc^mbn1^*, and cDNA was synthesized while using a random primer. We performed qRT-PCR analysis using the cDNAs to quantitate the total amount of the mRNAs for histones H1, H2A, H2B, H3, and H4. In every case, we observed that these mRNA levels were reduced to 30–40% of the normal control levels (Figure 5a). We further confirmed the results by assessing the levels of histone H4 mRNA in the LGs using RNA in situ hybridization, as it was difficult to perform qRT-PCR using RNAs prepared from LGs (Figure 5c,d). We observed a strong in situ hybridization signal of the histone H4 mRNA in 10% of the control LG cells on average (685 cells/6640 cells examined) (Figure 5c). They corresponded to S phase cells in which the histone genes are transcribed in a replication-dependent manner. In contrast, we observed that the signal of the histone H4 mRNA decreased to 20% of that in the control cells and the intensity of the RNA in situ signal also notably reduced in the LG of cells *mxc^mbn1^* (Figure 5e).

Furthermore, we investigated whether another HLB function, the processing of the pre-mRNAs of canonical histones, was also compromised in the mutant larvae. In normal cells, the processing factors in HLB cleave the pre-mRNA before the poly(A) addition signal to generate mature mRNAs lacking poly(A) tails. Therefore, only a small amount of histone mRNAs with poly(A) tails exist in normal cells [46]. We isolated the total RNAs from the third instar larvae of control (*w*) and *mxc^mbn1^* larvae and, subsequently, cDNA was synthesized from mRNAs with poly(A) tails while using oligo(dT) primers. Using the cDNAs, we performed qRT-PCR analysis to quantitate the levels of polyadenylated mRNAs for histones H1, H2A, H2B, H3, and H4. Consequently, the levels of all abnormal canonical histone mRNAs, with the exception of histone H1 mRNA, were two to three-fold higher than that of the normal controls (Figure 5b). From these observations, we concluded that a reduction in the levels of the total mRNAs of canonical histones and/or the production of abnormal polyadenylated mRNAs occurred due to impaired HLB activities in the *mxc* mutants.

### 2.7. Ectopic Overexpression of Polyadenylated mRNAs of Canonical Histones in CZ Reproduced the LG Hyperplasia Observed in the mxc Mutants

**Figure 6:**
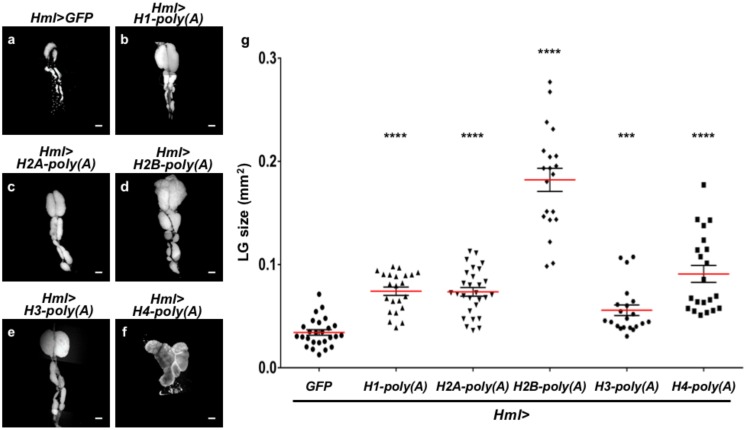
LG hyperplasia induced by ectopic expression of polyadenylated mRNA for each of canonical five histones. (**a**–**f**) LGs stained with DAPI prepared from mature third instar larvae having ectopic expression of polyadenylated mRNA for canonical histones that express in a DNA replication-dependent manner. A LG from control larvae (*Hml>GFP*) (**a**), LGs expressing polyadenylated *histone H1* mRNA (*Hml>HisH1-poly(A)*) (**b**)*,* expressing polyadenylated *histone H2A* (*Hml>HisH2A-poly(A)*) (**c**), expressing polyadenylated *histone H2B* (*Hml>HisH2B-poly(A)*) (**d**), expressing polyadenylated *histone H3* (*Hml>HisH3-poly(A)*) (**e**), expressing polyadenylated *histone H4* (*Hml>HisH4-poly(A)*) (**f**). (**g**) Quantification of LG size (*n* ≥ 21). (*** *p* < 0.001, **** *p* < 0.0001. Student *t*-test was used for comparison between control and *Hml>H1-poly(A)*. Welch’s *t*-test was used for other four comparisons). Note that ectopic overexpression of polyadenylated mRNA for *histoneH4* in CZ resulted in a significant hyperplasia of LG, as compared with the size of controls. Bars: 100 µm.

The observation that abnormal forms of canonical histone mRNAs were produced in the *mxc* mutants encouraged us to investigate whether the production of abnormal histone mRNAs can reproduce the LG hyperplasia that was observed in the mutants. We induced artificial histone mRNAs having poly(A) tails in CZ of normal LG to investigate this possibility. We performed ectopic expression of the histone mRNAs with poly(A) tails while using *Hml-Gal4* because we showed that the LG hyperplasia requires the loss of the *mxc* function in mature hemocytes. These artificial mRNAs were produced while using the 3′UTR sequences of other genes. We determined whether the ectopic expression affected the LG in normal larvae in the mature third instar stage (Figure 6). For example, the average size of LGs with ectopic expression of histone H2B polyadenylated mRNA was 5.3-fold higher than that of normal LGs from control larvae (*Hml>GFP*) at the same developmental stage (*n* = 20, *p* < 0.0001) (Figure 6a,d,g). We observed that several larvae harbored LGs that were >15 times larger than the average size of the control LGs (Figure 6g). Consistently, the LG size of larvae having mature hemocyte-specific expression of polyadenylated mRNAs for other four canonical histones (histone H1, H2A, H3, and H4) significantly increased in size (*p* < 0.001, *n* > 21) (Figure 6a–c,e–g). In summary, the ectopic expression of all five canonical histone mRNAs with poly(A)tails resulted in LG hyperplasia. In contrast, the mRNAs of non-canonical histones are originally transcribed as polyadenylated forms that are independent of DNA replication. The ectopic expression of mRNAs for the non-canonical histones, histone H3.3B and histone H2Av, did not affect the LG morphology and size (*n* > 10, *p* > 0.05 in Welch’s *t*-test). The DAPI-stained LG images were not presented. The results of this genetic analysis strongly suggested that the production of abnormal polyadenylated forms of canonical histone mRNAs was, at least partially, responsible for LG hyperplasia.

### 2.8. Ectopic Expression of the Negative Regulator Adgf-A, Which Suppresses Proliferation of Immature Hemocytes, and Signaling Factors Required for Induction of the Regulator Suppressed Hyperplasia in mxc^mbn1^

**Figure 7:**
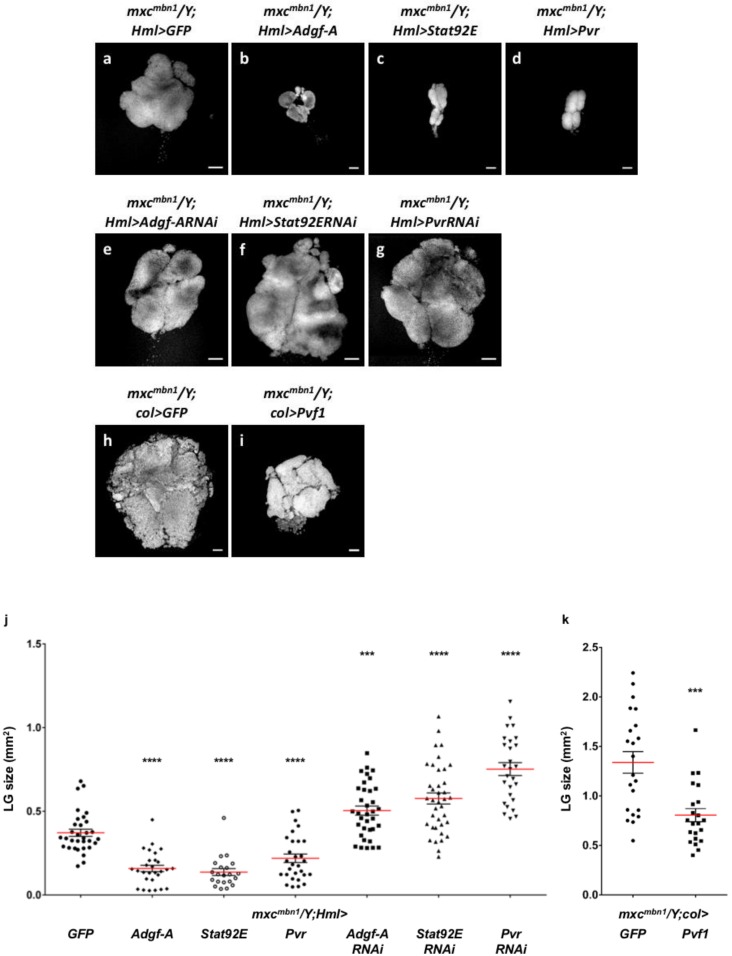
Suppression of the LG hyperplasia in *mxc^mbn1^* larvae having ectopic expression of *Adgf-A* and signaling factors required for its expression in the CZ. (**a**–**i**) DAPI-stained LGs prepared from *mxc^mbn1^* mature larvae at third instar stage. (**a**) A control LG expressing GFP in the CZ (**a**). (**b**–**d**) LGs having mature hemocyte-specific over-expression of *Adgf-A* (**b**), *Stat92E* (**c**), and *Pvr* (**d**). (**e**–**g**) LGs having mature hemocyte-specific expression of dsRNAs against mRNAs for *Adgf-A* (**e**), *Stat92E* (**f**), *Pvr* (**g**). (**h**,**i**) LGs having over-expression of GFP (**h**), and *Pvf1* (**i**) in PSC cells, from which Pvf1 is secreted. Bars: 100 µm. (**j**,**k**) Quantification of LG size. (*n* ≥ 21). *** *p* < 0.001, **** *p* < 0.0001. Student’s *t*-test was used for comparisons between *mxc^mbn1^/Y;Hml>GFP* and *mxc^mbn1^/Y;Hml>Adgf-A, mxc^mbn1^/Y;Hml>Stat92E, mxc^mbn1^/Y;Hml>Pvr*, *mxc^mbn1^/Y;Hml>Adgf-ARNAi.* Welch’s *t*-test was used for comparisons between *mxc^mbn1^/Y;Hml>GFP* and *mxc^mbn1^/Y;Hml>Stat92ERNAi, mxc^mbn1^/Y;Hml>PvrRNAi.* Note that ectopic overexpression of *Adgf-A*, *PvR*, and *Stat92E* in CZ resulted conversely in a significant suppression of the LG hyperplasia. Conversely, ectopic expression of dsRNAs against *Adgf-A*, *PvR*, and Stat92E mRNA in CZ resulted in a significant enhancement of the LG hyperplasia of *mxc^mbn1^*.

We demonstrated that thee loss of Mxc function in the CZ of LG is required for hyperplasia of the tissue in *mxc* mutants. It is known that Adgf-A is involved in the suppression of immature cell proliferation in LG, and this negative regulator is secreted from the CZ [37]. Combining these previous observations with our data, we speculated that Adgf-A might be involved in LG hyperplasia in *mxc^mbn1^* larvae. We next induced ectopic expression of this negative regulator in LGs of *mxc^mbn1^* to verify this hypothesis. The average LG size of *mxc^mbn1^* larvae having mature hemocyte-specific expression of Adgf-A (*mxc^mbn1^/Y; Hml>Adgf-A*) decreased by 42.6% when compared to that of *mxc^mbn1^* (*mxc^mbn1^/Y; Hml>GFP*) (*n* = 28, *p* < 0.0001 in Student’s *t*-test) (Figure 7a,b,j). It is also known that the expression of Adgf-A is induced by Pvf1, a signaling pathway that is mediated by Pvr (a Pvf1 receptor), and its downstream factor, Stat92E. We enhanced the Pvr-mediated signal that induces Adgf-A expression by overexpressing Pvr and Stat92E in CZ using the Gal4/UAS system to confirm that Adgf-A is involved in LG hyperplasia. When compared to that in *mxc^mbn1^* (*mxc^mbn1^/Y; Hml>GFP*), the average size of LG overexpressing Pvr or Stat92E in *mxc^mbn1^* (*mxc^mbn1^/Y;Hml>Pvr, mxc^mbn1^/Y;Hml>Stat92E*) significantly decreased (59.1% and 36.8% of that of the control (*n* = 21, *p* < 0.0001 for each) (Figure 7c,d,j). Conversely, we also investigated whether the depletion of Adgf-A, Pvr, and Stat92E in CZ enhanced LG hyperplasia (Figure 7a,e–g,j). All the larvae with ubiquitous expression of dsRNA against each of these three mRNAs raised at 25 °C died before the third instar stage. Thus, we were unable to perform qRT-PCR experiments to confirm whether the *UAS-RNAi* stocks can deplete the relevant endogenous mRNAs efficiently. However, as no off-targets against the dsRNA sequences that were expressed by these *UAS-RNAi* constructs have been identified, it is highly possible that the lethality is due to an efficient depletion of each mRNA in the larvae. Subsequently, we induced each of these dsRNAs in CZ (*Hml>Adgf-ARNAi, Hml>PvrRNAi,* and *Hml>Stat92ERNAi*). Consequently, the ectopic expression of dsRNA for Adgf-A, as well as that for Pvr and STAT92E in CZ, significantly enhanced LG hyperplasia in *mxc^mbn1^* (1.4 to 2.0-fold enlargement, *n* > 27, *p* < 0.001 to 0.0001 in Student’s *t*-test or Welch’s *t*-test). We induced the overexpression of Pvf1 in PSC, from which Pvf1 is secreted (*col>Pvf1*) (Figure 7h,i). We demonstrated that Pvf1 overexpression also significantly suppressed LG hyperplasia (by 60% of the original LG size in *mxc^mbn1^* larvae at the mature stage, *n* = 22) (*p* < 0.001 in Welch’s *t*-test) (Figure 7k). Based on these genetic data, we concluded that the downregulation of Adgf-A and Pvr-mediated signaling is required for the expression of factors that are involved in LG hyperplasia in *mxc^mbn1^* larvae, although direct evidence showing the down-regulation is lacking.

## 3. Discussion

### 3.1. Reduction of mxc Expression or Its Function in Mature Hemocytes Resulted in LG Hyperplasia Caused by Over-Proliferation of Undifferentiated Cells in the Tissue

Unique mutations that cause amino acid substitutions, such as *Ras^V12^*, and the production of fusion proteins, for example PML-RARα generated via T(15;17)(q22;12) translocation, have been identified as candidates for gene aberration responsible for the diseases in several types of human leukemia and lymphoma cells [47,48,49]. These mutations were proven to act in a dominant manner, and this results in malignant transformation of hematopoietic cells. Further, a number of recessive mutations in the tumor suppressor genes, such as *p53*, *Myc*, and the cyclin-dependent kinase inhibitor INK4 family genes, are also deeply implicated in human leukemia and lymphoma [4,50,51]. On the other hand, *mxc^mbn1^*, which is responsible for the malignant hyperplasia of immature cells in LG, is a recessive and loss of function mutation [20,22] and this study. We concluded that a reduction in *mxc* expression or its function in mature hemocytes is responsible for LG hyperplasia in the *mxc* mutants. It is likely that a reduction, but not a complete loss of the gene function, is essential for LG malignant hyperplasia, as the gene product plays a critical role in histone biogenesis. A previous study has demonstrated that the over-proliferation of undifferentiated hemocyte precursors, but not mature hemocytes, in LG resulted in hyperplasia of the tissue [23]. These phenotypes of the *mxc^mbn1^* mutants were reminiscent of symptoms of human leukemia and lymphoma [52,53]. Immature hemocyte precursors that are present in LG are arrested at the G_0_ state of the cell cycle in the tissue. The maintenance of the quiescent state is required for a certain signal emanating from the mature hemocytes to control the proliferation of the immature cells in LG [37,54]. While considering these previous findings with the current genetic data, we speculated that the reduced expression or function of *mxc* in mature hemocytes resulted in a failure to maintain the quiescent state of the immature hemocyte precursors, and this eventually led to malignant hyperplasia of the LG.

Along with the disappearance of the first lobe in *mxc^mbn1^* mutants, hyperplasia of second and third lobes was frequently observed in the LGs. The first lobe, but no other posterior lobes, possesses mature hemocytes in the CZ and PSC, which acts as a stem cell niche. Similar LG phenotypes, such as excess enlargement, and thereby rupture of the first lobe of LG, were also observed in the *Ubc9* mutant larvae [55]. A large number of hemocytes have to be produced in LGs, not only in these mutants, but also in the cases of severe immune challenge, such as severe bacterial infection or oviposition of wasp. Consequently, this is sometimes accompanied by enlargement and the resultant burst of the first lobe [56]. In both cases, excessive hemocytes were released into hemolymph and hyperplasia of the second and third lobes was observed. Upon elimination of the first lobes, the signal suppressing proliferation of undifferentiated cells was lost as a result. Therefore, suppression of precursor cell proliferation can be avoided and, subsequently, the proliferation of immature cells in the posterior lobes can be stimulated. We observed de novo generation of PSC and CZ in LGs, in which the first lobes were eliminated in the mutant larvae. The presence of the PSC and CZ in the posterior lobes of LGs lacking the first lobes has not been analyzed in *Ubc9* mutants or larvae with severe infection in the LGs. Therefore, we cannot conclude whether the *de novo* production of PSC cells and mature hemocytes is a backup system originally provided or characteristic of the *mxc* mutants. Whether the signaling center, including a stem cell-niche and mature hemocytes, can be newly generated when the first lobe is lost warrants further investigations.

### 3.2. Mature Hemocyte-Specific Expression of Polyadenylated mRNAs for Canonical Histones Was Responsible for Hyper-Proliferation of Immature Cells in LG Hyperplasia

HLB is a nuclear body that is associated with the histone gene cluster and it is required for transcription of genes for five canonical histones in the S phase. It is also required for 3′ processing to produce mRNAs without poly(A) tails [13,43,57]. Both reduced the levels of mRNAs for the canonical histones and the production of abnormal histone mRNAs harboring poly(A) tails were observed in *mxc^mbn1^* larvae. Ectopic expression of the abnormal type of each histone mRNA was sufficient for the reproduction of LG hyperplasia. Therefore, generation of polyadenylated mRNAs is a more likely cause of LG hyperplasia in *mxc^mbn1^*. However, we cannot exclude the possibility that a reduction in mRNA levels led to hyperplasia. The mRNAs cannot be readily depleted sufficiently via dsRNA overexpression while using the Gal4/UAS system, as large amounts of the histone mRNAs are expressed from more than 100 sets of histone genes in the *Drosophila* genome [45].

The histone genes are transcribed in a DNA replication-dependent manner and their mRNAs exclusively exist in S phase cells [41,58]. Most of the mRNAs do not possesses poly(A) tails, owing to the action of HLB. In fact, histone mRNAs with artificial poly(A) tails exist at all cell cycle stages, not being restricted to the S phase [59]. The replication-coupled synthesis of histones can be also achieved as a consequence of selective degradation of the mRNAs before the end of the S phase [46]. The S phase-specific expression of canonical histones requires the rapid degradation of the mRNAs at the end of the S phase [60]. In contrast, the degradation of polyadenylated mRNAs is usually time-consuming, as it occurs via multiple steps, including processes that remove the poly(A) tails from the mRNAs [61]. The impediment of replication-dependent synthesis of histones can reduce the amount of total core histones. In addition, the poly(A) tails participate in nuclear export of mRNA toward the cytoplasm [62]. The nuclear export of mRNAs is mediated by the TAP protein that is associated with the poly(A) tails of mRNAs [63]. On the other hand, the mRNAs for canonical histones originally lacking poly(A) tails are associated with PHAX proteins, and the RNA-protein complexes are exported from the nucleus [64]. The alteration of the nuclear export system due to aberrant poly(A) addition on the histone mRNAs might prevent the RNA transport and translation required for efficient production of core histones.

The formation of rigid chromatin structure is certainly interrupted, when replication-coupled incorporation of core histones is inevitably affected. Thus, it is highly possible that the production of polyadenylated histone mRNAs affected the chromatin structure, which eventually affected gene expression in the LG cells. Some studies have shown that reduced transcription and/or expression of histone genes resulted in gross alteration of gene expression patterns in human cells [65,66]. A previous RNA-seq analysis revealed that the expression of several genes, such as tumor-related genes, such as nimC1 and Pvf2, related to tumorigenesis or cell cycle progression, was altered in the *mxc^mbn1^* larvae [23]. Consistently, earlier studies have reported that hypomorphic *mxc* mutations enhanced phenotypes of *PcG* mutants and cause ectopic expression of homeotic genes [18,19]. On the basis of these results, we speculated that modified chromatin structure due to reduced histone expression in the *mxc* mutants altered the expression of the genes that maintain immature cells in the quiescent stage. It is also possible that these alterations eventually led to malignant hyperplasia of LG cells in *mxc^mbn1^*. Hence, the identification of the altered gene expression responsible for malignant LG hyperplasia in the *mxc* mutant is important.

Genome instability and histone expression levels show positive correlation [67]. A previous study on a hypomorphic *mxc* mutant reported that the mRNA levels of four canonical histones, with the exception of histone H3, decreased, while the level of histone H3 increased in other hypomorphic *mxc* mutants [24]. Although the mechanism underlying the changes in histone gene expression has not yet been clarified, it can be interpreted, as follows; the alterations in the expression of the five histone mRNAs resulted in DNA replication stress and the accumulation of DNA damage [24,68]. However, another study showed that the mRNA levels of the five canonical histones were constantly reduced in the testes of the hypomorphic mutants for *mxc* [25]. In addition, DNA damage foci that were examined by immunostaining using anti-H2Av antibody were not evident in the mutant cells. Further studies regarding DNA replication stress in the *mxc* mutants are necessary.

### 3.3. Involvement of mxc in the Signaling That Induces Adgf-A Expression, Which Suppresses Excess Proliferation of Immature Cells in LG

The reports show that the CZ in LG is rich in mature hemocytes, which transmit a signal to suppress the excessive proliferation of immature hemocyte precursors [37,54]. The signal corresponds to an extracellular protein, called Adgf-A, which is expressed in mature cells that are located in the CZ. The factor is secreted from the mature hemocytes in LG and acts on immature precursors of hemocytes in the MZ to suppress their proliferation. Adgf-A expression requires the activation of a signaling pathway that is mediated by Pvr, which is homologous to a receptor for a mammalian PDGF and VEGF ligands. For the activation of the signaling pathway, a ligand for Pvr, called Pvf-1, and a signaling molecule, called Stat92E, acting downstream of Pvr, are required [54]. Here, we showed that the ectopic expression of Adgf-A and enhancement of Pvr-Stat92E-mediated signaling resulted in the suppression of LG hyperplasia In mature hemocytes of the *mxc^mbn1^* LG. Conversely, the depletion of Adgf-A and downregulation of the Pvr-Stat92E-mediated signaling pathway enhanced tissue hyperplasia. These genetic interactions between Mxc and Adgf-A, or that between Mxc and the signaling molecules, strongly suggest that *mxc* is closely related to an Adgf-A expression or signal transduction mediated by Pvr and Stat92E. While considering that the supply of core histones required for generating the substantial chromatin structure decreases in the LG cells of the *mxc* mutants, expression Adgf-A, or of signaling factors that are essential for *Adgf-A* expression, was downregulated due to altered chromatin structure in the mutant LG cells. To verify this hypothesis, it will be important to show that Adgf-A expression decreases in the mature hemocytes in the CZ of the mutant LG. Furthermore, whether Mxc or Mxc-HLB colocalizes with Stat92E in the LG nuclei warrants further investigation. Overall, we propose that the loss of HLB function and thereby the inhibition of canonical histone mRNA expression that is required for substantial chromatin construction, is critical for malignant LG hyperplasia in this *Drosophila* leukemia model. Our findings in the *Drosophila* model will enable us to consider a similar involvement of NPAT, the human counterpart of Mxc, in the pathogenesis and development of leukemia. Animal models of other species should clarify the hypothesis derived from investigations while using *Drosophila*.

## 4. Materials and Methods

### 4.1. Drosophila Stocks

All of the *Drosophila melanogaster* stocks were maintained on standard cornmeal food at 25 °C, as previously described [69]. Gal4-dependent expression was performed at 28 °C. *w^1118^* (*w*) was used as a normal control stock. Recessive lethal alleles of *mxc* showing the LG tumor phenotype, *mxc^mbn1^* (#6360) and *mxc^16a-1^* (#7133) were obtained from Bloomington Drosophila Stock Center (BDSC) (Bloomington, IN, USA) [23]. Non-tumorous hypomorphic allele, *mxc^G43^* and *mxc^G46^*, which are able to develop to adult stage and pharate adult stage, respectively, were obtained from BDSC [25]. Amorphic allele, *mxc^G48^* was also obtained from BDSC (#7141). For the depletion of the *mxc* mRNA, *P*{*TRiP.HMS00444*} (*UAS-mxcRNAi*) was used. This stock (#32446 from BDSC) can be used for efficient Gal4-dependent depletion of the gene [23]. To rescue the phenotypes of *mxc* mutants, we used *PBac*{*Ubi-GFP-Mxc*} (*Ub-Mxc*) [16] (a gift from Dr. R.J. Duronio (North Carolina Univ., Chapel Hill, NC, USA). The following Gal4 driver stocks were used for ectopic expression in specific larval tissues; *P*{*w^+mC^=Hml-GAL4.G*}*6-4P* (*Hml-Gal4*) for the induction of the gene expression in CZ of LG (a gift from Dr. D. Hultmark (Umea Univ., Umeå, Sweden)), *P*{*upd3-Gal4*}(*upd3-Gal4*) for the induction in MZ of LG (a gift from Dr. N. Perrimon (Harvard Medical School, Boston, MA, USA)), *P*{*col-Gal4*}(*col-Gal4*) for the induction in PSC of LG [70] (a gift from Dr. M. Crozatier (Université Toulouse III, Toulouse, France)), and *p{Actin5C-Gal4}25F01* (*Act-Gal4*) for ubiquitous expression (#4414 from BDSC). For a depletion of other HLB components, we used the following *UAS-RNAi* stocks; *P*{*TRiP.HMS00364*} (*UAS-Spt6RNAi*) (#32373), and *P{TRiP.GL01166}* (*UAS-Cpsf160RNAi*) (#42478), both which were obtained from Bloomington Drosophila Stock Center. *P*{*KK104734*}*VIE-260B* (*UAS-muteRNAi*) (#105257), *P*{*KK102041*} (*UAS-Adgf-ARNAi*) (#110152), *P*{*KK100519*} (UAS-Stat92ERNAi) (#106980), *P*{*GD14375*} (*UAS-PvrRNAi*) (#43459) from Vienna Drosophila RNAi Center. For ectopic expression of canonical histone mRNAs carrying a poly (A) tails at the 3′ends, we used the following stocks; *UAS-HistoneH1-YFP* [71] (a gift from Dr. A. Tulin (Univ. North Dakota, Grand Forks, ND, USA)), *UAS-HistoneH2A-YFP* [71] (a gift from Dr. A. Tulin (Univ. North Dakota)), *UAS-HistoneH2B-GFP* [72] (a gift from Dr. Xin Chen (Johns Hopkins Univ., Baltimore, MD, USA), *UAS-HistoneH3-GFP* [72] (a gift from Dr. Xin Chen (Johns Hopkins Univ.), and *UAS-histone H4 ORF-3 x HA^F000777^* (*UAS-HistoneH4-HA*) obtained from Fly ORF. *UAS-HintoneH2Av* (#5941 from BDSC). The mRNAs that are induced by these UAS stocks possess 3′UTR sequences and poly(A) tails of other genes after each of coding sequences and a fluorescence tag.

For the ectopic expression of Adgf-A and signaling factors that induce the factor, the following UAS stocks were used; *PBac{WH}Adgf-A^f04691^* (*UAS-Adgf-A*) (#04691 from Exelixis at Harvard Medical School), *M{UAS-Stat92E.ORF.3xHA}ZH-86Fb* (*UAS-Stat92E*) (#F000750 from FlyORF(Univ. of Zurich, Zurich, Switzerland)), *M{UAS-Pvr.ORF.3xHA}ZH-86Fb* (*UAS-Pvr*) (#F000896 from FlyORF), and *M{UAS-Pvf1.ORF.3xHA.GW}ZH-86Fb* (*UAS-Pvf1*) (#F002862 from FlyORF).

### 4.2. LG Preparation

Normal controls (*w/Y*) pupated at six days (28 °C) and seven days (25 °C) after egg laying (AEL), whereas some of the *mxc^mbn1^* mutant remained in third instar larval stage at eight days (28 °C) and 10 days (25 °C) AEL. The comparative analysis of hemizygous mutants and controls was performed on the same day, when the wandering third instar larval stage was seen, to minimize the possibility of a delay that might allow the hyperplastic tissue to grow. Alternatively, the tissues were collected from hemizygous mutant larvae one day after the timing of the LG collection from control larvae. For the staging of the larvae, parent flies were transferred into a new culture vial and left there to lay eggs for 24 h. Careful attention was given to avoid overcrowding of the larvae in the vial. A pair of anterior lobes of the LG without connected cardiac cells from mature stage larvae were isolated and fixed with 3.7% formaldehyde for 15 min. to compare the size of LGs. The fixed samples were mildly flattened under constant pressure while using an apparatus so that the tissue became spread out into cell layers with a constant thickness. The LG area of each DAPI-stained sample was measured while using ImageJ ver.1.47 (https://imagej.nih.gov/ij/).

### 4.3. qRT-PCR Analysis

The total RNA was extracted from third instar larvae with each genotype while using the Trizol reagent (Thermo Fisher Scientific, Waltham, MA, USA). cDNA synthesis from the total RNA was carried out using the PrimeScriptTM High Fidelity RT-PCR Kit (TaKaRa, Clontech Laboratories, Shiga, Japan) using an oligo dT primer. Real-time PCR was performed while using the FastStart Essential DNA Green Master (Roche, Mannheim, Germany) and a Light Cycler Nano instrument (Roche). According to software the Primer3Plus (http://www.bioinformatics.nl/cgi-bin/primer3plus.cgi), the primers for qRT-PCR were synthesized as follows: RP49-Fw,5′-TTCCTGGTGCACAACGTG-3′; RP49-Rv,5′-TCTCCTTGCGCTTCTTGG3′; histoneH1-FW,5′-AGTTGCAACGTCCGCTTC-3′; histoneH1-RV,5′-TTGTGCCAGCAGATCCAG-3′; histoneH2A-Fw,5′-GAAGGGAAACTACGCAGAGC-3′; histoneH2A-Rv,5′-AGCCAACTCGAGAACCTCAG-3′; histoneH2B-Fw, 5′-TTCGTCGAAGGCGATGAG-3′; histoneH2B-Rv 5′-CGAGCGCTTGTTGTAGTGAG-3′, histoneH3-Fw, 5′-GAGCACCGAGCTTCTAATCC-3′; histoneH3-Rv 5′- CTTCCTGCAGAGCCATAACC-3′, histoneH4-Fw, 5′-GCGTCATCGCAAAGTACTGC-3′; histoneH4-Rv; 5′-CCAGATATGCGCTTCACACC-3′ Each sample was duplicated on the PCR plate, and the final results average three biological replicates. For the quantification, the ∆∆Ct method was used to determine the differences between target gene expressions relative to the reference *Rp49* gene expression.

### 4.4. Lymph Gland Immunostaining

For the immunostaining of larval lymph glands, anterior lobes of LGs were dissected from matured third instar larvae and fixed in 3.7% paraformaldehyde in PBS for 15 min. at 25 °C. After repeated washing, samples were blocked with PBS containing 0.1% Triton X-100 and 10% normal goat serum and the fixed samples were incubated with primary antibodies at 4 °C overnight. The following antibodies were used as primary antibodies: anti-Mxc antibody [16] (a gift from Dr. B. Marzluff, diluted at 1: 2000), anti-Lsm11 antibody [73] (a gift from Dr. J. Gall, 1: 1000). After extensive washing, specimens were incubated with Alexa 594 or Alexa 488 secondary antibodies (1: 400; Molecular Probe, Eugene, OR, USA). The LG specimens were placed on a fluorescence microscope (Olympus, Tokyo, Japan, model: IX81), which was outfitted with excitation, emission filter wheels (Olympus). The fluorescence signals were collected while using 10×, 20×, 40× dry objective lens. The specimens were illuminated with UV filtered and shuttered light while using the appropriate filter wheel combinations through a GFP filter cube. GFP fluorescence images were captured with a CCD camera (Hamamatsu Photonics, Shizuoka, Japan). Image acquisition was controlled through the Metamorph software version 7.6 (Molecular Devices, Sunnyvale, CA, USA) and processed with ImageJ or Adobe Photoshop CS (Adobe Systems, San Jose, CA, USA).

### 4.5. Fluorescence In Situ Hybridization (FISH)

We amplified a 351 bp-long genomic DNA of the gene while using a set of a PCR primer, 5′-TACTCGAGCTTTCGTGCTGTGCGTG-3′ and 5′-CGGAATTCTAACCGCCAAATCCGTA-3′, for RNA in situ hybridization to examine a distribution of *histoneH4* mRNA in larval LGs. The DNA fragment was inserted into *pOT2* plasmid to produce a RNA probe for the FISH using FISH Tag RNA Kit (Catalogue #F32954 Invitrogen, Carlsbad, CA, USA). The LGs were collected from third instar larvae at mature stage and fixed with 4% paraformaldehyde. Subsequently, they were treated with 80% acetone for 10 min. at −30 °C, rehydrated in PBS-10% Triton X-100 (PBST), and then fixed again with 4% paraformaldehyde. RNA hybridization was carried out with fluorescence-labelled RNA probe described above in a hybridization buffer provided at 56 °C, for 16 h following the manufacturer’s instructions. After repeated rinse steps, the LG samples were observed while using a confocal microscope (Fv-10i, Olympus, Tokyo, Japan).

### 4.6. Statistics

The results of the LG area measurements were presented as bar graphs or scatter plots created using GraphPad Prism 6. The area in pixels was calculated and an average determined for each LG. Each single dataset was assessed while using Welch’s *t*-test or Student’s *t*-test, as described (Araki et al., 2019). An *F*-test was performed to determine equal or unequal variance. If the value was less than 0.05, and then P-values were calculated using Welch’s *t*-test of unequal variance. If the *F*-value was greater than 0.05, then the P-values were calculated while using the Student’s *t*-test of equal variance. Statistical significance is described in each figure legend: *p < 0.05, ** *p* < 0.01, *** *p* < 0.001 and **** *p* < 0.0001.

## 5. Conclusions

We performed genetic analysis of *Drosophila* mutants showing malignant hyperplasia in larval hematopoietic tissues to reveal the mechanisms by which the leukemia-like phenotypes appear. Reduced activity of the *mxc* gene encoding a component of the HLB essential for histone mRNAs in mature cells of the LG is responsible for the hyper-proliferation of immature cells in the mutant LG. A loss of HLB function, especially 3′-end processing of histone mRNAs, is critical for the malignant LG hyperplasia. It is likely that that *mxc* is involved in the regulation to induce Adgf-A, which suppresses immature cell proliferation in LG. We propose that the inhibition of the suppression mechanism by the *mxc* mutation is involved in the LG hyperplasia in this leukemia model in *Drosophila*.

## Supplementary Materials

Supplementary Materials can be found at https://www.mdpi.com/1422-0067/21/5/1586/s1.

## Abbreviations

HLB	Histone locus body
LG	Lymph gland
CZ	Cortical zone
MZ	Medulla zone
PSC	Posterior Signaling Center
PC	Pericardial cells
Adgf-A	Adenosine deaminase-related growth factor-A
