# Supplementary material for: Loss of Histone Locus Bodies in the Mature Hemocytes of Larval Lymph Gland Result in Hyperplasia of the Tissue in mxc Mutants of Drosophila
Source: Int J Mol Sci. 2020 Feb 26;21(5):1586. doi: 10.3390/ijms21051586 (PMC7084650; doi:10.3390/ijms21051586)
Supplement: Supplementary file 1 [file ijms-21-01586-s001.pdf]

Supplementary Materials:

Loss of Histone Locus Bodies in the Mature Hemocytes of Larval Lymph Gland Result in Hyperplasia of the Tissue in *mxc* Mutants of *Drosophila*

Masanori Kurihara †, Kouyou Komatsu †, Rie Awane and Yoshihiro H. Inoue *

Department of Insect Biomedical Research, Center for Advanced Insect Research Promotion, Kyoto Institute of Technology, Matsugasaki, Sakyo-ku, Kyoto 606-0962, Japan; masa.megane.masa@gmail.com (M.K.); yoshikouyou@gmail.com (K.K.); r1a.awa@gmail.com (R.A.)

* Correspondence: yhinoue@kit.ac.jp; Tel.: +81-75-7247876

† These authors contributed equally to this work.

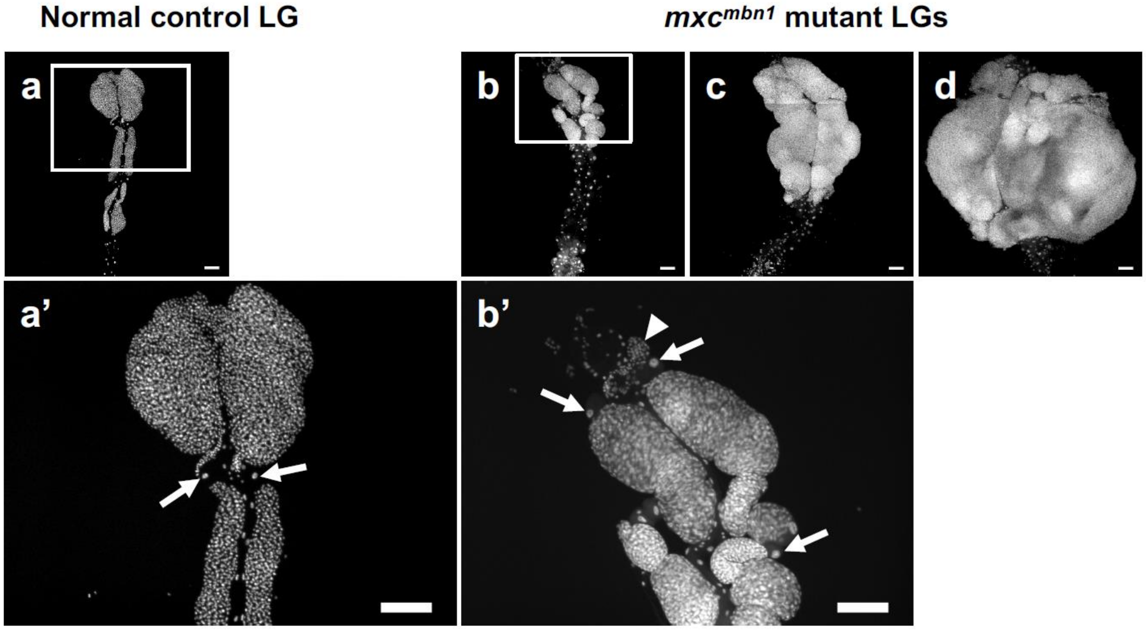

Figure. S1. Ruptured hyperplasic lobes at the anterior end of lymph gland and a progression of overgrowth of the posterior lobes according to larval development at 3rd instar stage. (**a**–**c**) Fluorescence micrographs of DAPI-stained LGs prepared from 3rd instar larvae at mature stage. (**a**,**a′**) A LG from a control male larvae at 7 days after egg-laying (*w*). (**a′**) an enlarged image of the most anterior region of a LG, surrounded by a white rectangular frame. (**b**–**d**) LGs from mxc^{mbn1} male larvae according to development of 3rd instar stage. (**b**,**b′**) A LG from a mxc^{mbn1} male larvae at 7 days after egg-laying. (**b′**) an enlarged image of the most anterior region of a LG, surrounded by a white rectangular frame. Arrows indicate PC cells which are originally located between lobes. Arrowhead points remaining cells that were a part of the original first lobe (currently lost in this LG). (**c**,**d**) LGs from mxc^{mbn1} male larvae at 10 days after egg-laying (**c**) and at 13 days after egg-laying (**d**). Bars: 100 μm.

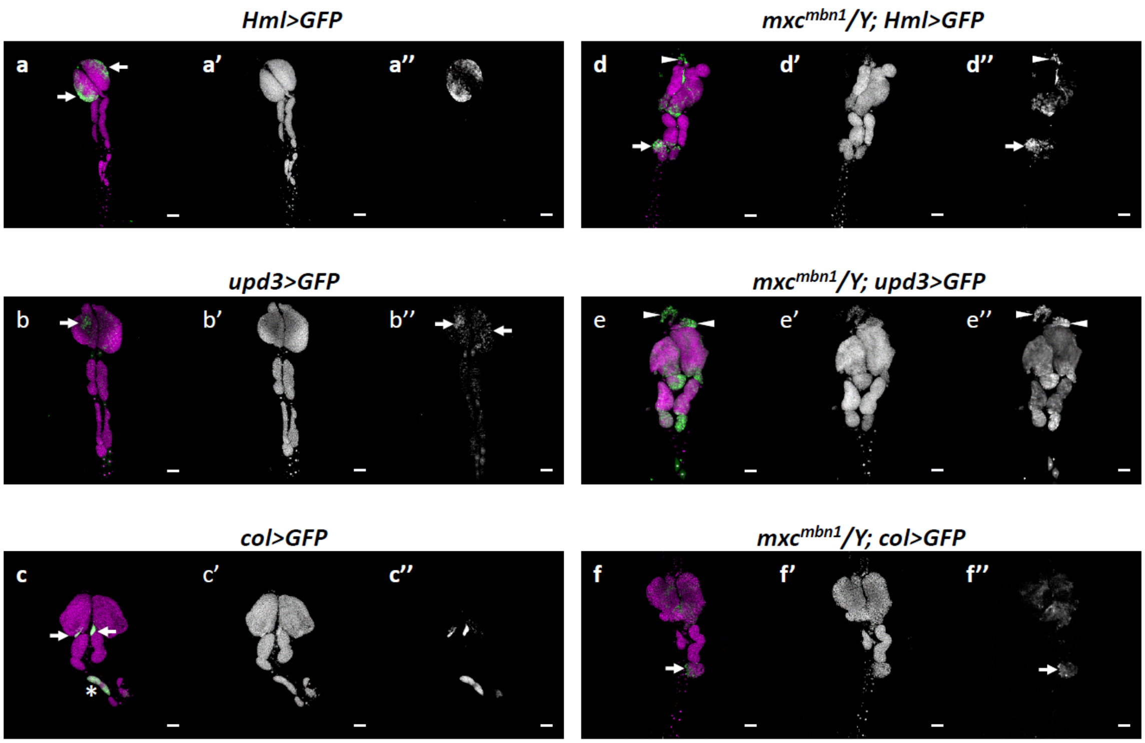

Figure S2. Appearance of cells expressing a marker for mature hemocyte and those expressing a PSC cell marker in the posterior lobes in larval LG lacking the original 1st lobe in *mxc^{mbn1}* larvae. (**a**–**f**) Distribution of GFP-expressing cells in LG from 3rd instar larva at mature stage. (**a**,**d**) The mature hemocytes in LG are labelled by *Hml>GFP*. (**b**,**e**) The immature hemocyte precursors in LG are labelled by *upd3>GFP*. (**c**,**f**) PSC cells are labelled by *col>GFP*. A LG from normal control LG (**a**–**c**), and from *mxc^{mbn1}* mutant larvae (**d**–**f**) at mature 3rd instar stage. The *mxc^{mbn1}* mutant LGs in d and e are lacking the 1st lobe which was localized at the anterior end of the LGs. Arrows in a indicate CZ. The arrow in d and d″ indicate de novo CZ contained mature hemocytes abundantly. The arrowhead in d and d″ indicates GFP positive cells (*Hml>GFP*) corresponding to remaining CZ cells of the 1st lobe at the anterior end. The arrow in b and arrows in b″ indicate MZ. Arrowheads in e indicates GFP positive cells (*upd3>GFP*) corresponding to remaining MZ cells of the 1st lobe at the anterior end. The arrows in c indicate PSC. The asterisk in c indicate cells labelled by *col>GFP*, which appears in a part of a low of PC cells. The arrow in f indicates de novo PSC cells in a posterior lobe. Magenta in a–f (white in **a′**–**f′**); DAPI staining. Green in a–f (white in **a″**–**f″**); GFP fluorescence. Scale bars: 100 μm.

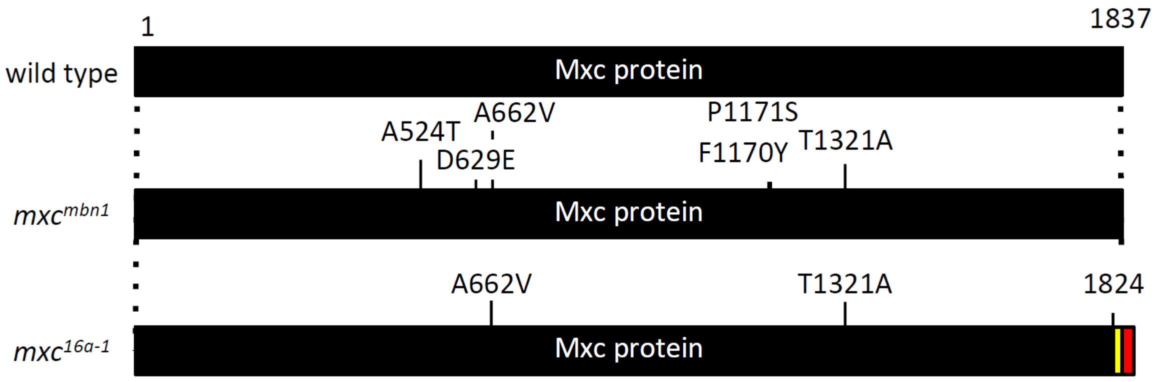

Figure S3. Amino acid substitutions observed in the *mxc* mutations showing the hyperplasia phenotype. Genomic DNA sequences of the mxc gene isolated from male larvae hemizygous for *mxc*^{mbn1} and those for *mxc*$^{16a\text{-}1}$ were determined and compared the predicted amino acid sequences with wild-type sequences appeared in the fly base (http://flybase.org/download/sequence/FBgn0260789/FBgn). We confirmed that a nonsense mutation occurred at the end of the coding region from *mxc*$^{16a\text{-}1}$, which resulted in a C-terminal truncation, as previously reported [16].
